# A Review of the Risk Factors Associated With Poor Outcomes in Patients With Coronavirus Disease 2019

**DOI:** 10.7759/cureus.10350

**Published:** 2020-09-10

**Authors:** Muhammad Hanif, Muhammad Adnan Haider, Qianlan Xi, Mukarram Jamat Ali, Muhammad Umer Ahmed

**Affiliations:** 1 Medicine, Khyber Medical College Peshawar, Hayatabad Medical Complex, Peshawar, PAK; 2 Internal Medicine, Allama Iqbal Medical College/Jinnah Hospital, Lahore, PAK; 3 Internal Medicine, West China Hospital, Sichuan University, Chengdu, CHN; 4 Medicine, King Edward Medical University, Lahore, PAK; 5 Internal Medicine, Ziauddin University, Karachi, PAK

**Keywords:** covid-19, sars-cov-2, risk factors, co-morbidities, predictors, diabetes mellitus, hypertension

## Abstract

The global pandemic of coronavirus disease 2019 (COVID-19) and its rapid spread throughout the globe is of much concern. With little known about the peculiar virus and the changing mortality and morbidity, we attempt to review the risk factors associated with significant outcome.

We conducted a review of the information available in medical journals published on COVID-19 risk factors associated with poor outcomes using PubMed®, Google Scholar, and material published online. The risk factors associated with poor outcome were kept in particular consideration. A total of 96 articles were thoroughly reviewed and analyzed so as to highlight the risk factors and the subsequent disease presentation that were present in patients with COVID-19. With little data available in this regard, emphasis and consideration of risk factors might help health care workers preclude the worst outcome. From the aforementioned search we can conclude that the most prevalent risk factors were reported to be hypertension followed by diabetes. In terms of mortality, age greater than 65 was the most significant risk factor. Among non-survivors, coagulation profile including d-dimers, prothrombin time, and inflammatory markers like erythrocyte sedimentation rate (ESR), C-reactive protein (CRP), and serum ferritin levels were very deranged. Much emphasis and consideration in relation to risk factors must be deliberated by health care workers so as to prevent severe outcomes and mitigate appropriate treatment modalities.

## Introduction and background

Coronavirus disease (COVID-19) caused by the severe acute respiratory syndrome coronavirus 2 (SARS-CoV-2), initially began in Wuhan, China and now has been declared a global pandemic by the World Health Organization on March 11th, 2020 [1,2]. As the COVID-19 pandemic rages on (May 2020), there is an urgent need for clinical and laboratory predictors of severe and fatal disease. Here, we review the possible predictors of mortality for COVID-19 to help clinical professionals in the stratification of patients at an enhanced risk of developing severe disease and to discriminate between those at high or low risk of mortality.

## Review

The various predictors of mortality associated with COVID-19 are shown in Table 1 and Table 2 and discussed below.

**Table 1:** Illustrates the predictors of mortality in patients with coronavirus disease 2019 (COVID-19)

Sr. #	PREDICTORS
1.	Age
2.	Hematologic Biomarkers
3.	Biochemical markers
4.	Inflammatory biomarkers
5.	Coagulation profile
6.	Cerebrovascular Co-morbidities
7.	Hypertension
8.	Diabetes Mellitus

**Table 2:** Summarizes the predictors of mortality in patients with coronavirus disease 2019 (COVID-19)

PREDICTORS	AUTHORS	STUDY DESCRIPTION	STUDY TYPE/GEOGRAPHY
AGE	1.Yang X, et al. 2.Mehra MR, et al. 3.Hu L, et al. 4.Du RH, et al. 5.Wang K, et al. 6.Wang K et al. 7.Nikpouraghdam M, et al. 8.Leung C, et al. 9 Martins-Filho PR, et al.	1. COVID-19 non-survivors were older in comparison to survivors. 2. Age older than 65 years was associated with an increased risk of death in COVID-19 patients i.e. increased mortality by 10% with odds ratio (OR) 1.93. 3. Age over 65 predicted unfavorable clinical outcomes including death with OR=3.549 AND p<0.001. 4. Mortality due to COVID-19 pneumonia was concentrated in people ages ≥65 years. 5. The mean age of the non-survivor group i.e. 65.6±12.6 was remarkably higher than the mean age of the survivor group 46.0±14.4 years. 6. Higher hazard ratio (HRs) or 15-day in-hospital death were associated with age older than 65 years i.e. HR 1.962. 7. For a one-year increase in the age, the odds of death in patients suffering from COVID 19 increased significantly i.e. OR=1.05 8. Deceased had mean age of 75 years in comparison to survivors i.e. mean age 68 years, p=0.017. 9. Increased risk for in-hospital death in older patients i.e. MD=13.8 years.	1.Retrospective, observational study/China. 2.Retrospective, observational study/Asia, Europe and North America. 3.Retrospective study/China. 4.Multi-center observational study/China. 5.Retrospective cohort study/China. 6.Observational cohort study/China. 7.Retrospective study/Iran. 8.Retrospective study/China. 9.Retrospective study/China.
HEMATOLOGICAL BIOMARKERS	1.Ruan Q, et al. 2.Zhou F, et al. 3.Wang K, et al. 4.Hu L, et al. 5.Wang K, et al. 6.Liu Y, et al. 7.Henry BM, et al. 8.Martins-Filho PR, et al.	1. Significant differences exist in white blood cell (WBC) counts, absolute values of lymphocytes, platelets between non-survivor group and survivor group. 2. Significantly higher count in baseline lymphocyte in survivors than non-survivors. 3. Severe lymphopenia was associated with increase mortality in COVID-19 patients. 4. Leukocytosis and neutrophilia predicted unfavorable clinical outcomes in COVID-19 patients with p<0.001. 5. WBCs, specifically neutrophils, were considerably higher whereas lymphocytes were remarkably lower in the non-survivor group as compared to the survivor group. 6. There was an 8% higher risk of in-hospital mortality for each unit increase in neutrophil to lymphocyte ratio (NLR) and the patients in the highest NLR tertile had a 15.04-fold higher risk of death. 7. COVID-19 patients who had fatal outcomes were noted to have a significantly increased WBC count and a decreased amount of both, lymphocytes and platelets, as compared to those patients who survived. 8. COVID-19 non-survivor patients had increased levels of white blood cells and neutrophils in comparison to survivors.	1.Retrospective study/China. 2.Retrospective cohort study/China. 3.Retrospective study/China. 4.Retrospective study/China. 5.Observational cohort study/China. 6.Retrospective cohort study/China. 7.Meta-analysis. 8.Retrospective study/China.
BIOCHEMICAL MARKERS	1.Ruan Q, et al. 2.Zhou F, et al. 3.Wang K, et al. 4.Henry BM, et al. 5.Martins-Filho PR, et al. 6.Santoso A, et al.	1. Serum total bilirubin, blood urea nitrogen, serum creatinine (Cr), myoglobin and cardiac troponin were higher in non-survivors in comparison to survivors. 2. Cardiac troponin I, creatinine, alanine aminotransferase (ALT), and lactate dehydrogenase (LDH) were elevated in non-survivors compared with survivors throughout the clinical course and showed a significant increase with the progression of illness. 3. Creatinine, blood urea nitrogen (BUN), ALT, aspartate aminotransferase (AST), LDH, and blood ammonia were remarkably higher whereas GFR and serum albumin were significantly lower in the non-survivor group. 4. Non-survivor COVID-19 patients had significantly elevated cardiac troponin levels, liver enzymes (ALT and AST) and renal biomarkers (urea nitrogen, creatinine) at presentation. 5. Non-surviving patients had increased levels of creatinine, creatine kinase, hypersensitive cardiac troponin I, lactate dehydrogenase, and decreased level of albumin in comparison to survivors. 6. There was a strong association of cardiac injury with higher mortality of COVID-19 patients, defined as high sensitive cardiac troponin I>99^th^ percentile.	1.Retrospective study/China. 2.Retrospective cohort study/China. 3.Observational Cohort study/China. 4.Meta-analysis. 5.Retrospective study/China. 6.Meta-analysis.
INFLAMMATORY MARKERS	1.Rong-Hui DU et al. 2.Wang ZL, et al. 3.Zhou F et al. 4.Lui W, et al.	1. Non-survivors had high level of C-reactive protein 86.4mg/L^-1^ (39.9-105.5) compared to survivors 36.0mg/L^-1^ (19.3-91.0) with p=0.012. 2. SpO_2_< 90% was associated with a higher level of erythrocyte sedimentation rate (ESR) and C-reactive proteins (CRP) in comparison with the patients having SpO_2_≥ 90% (p<0.001). 3. Non-survivors had high level of interleukin 6, procalcitonin, serum ferritin, C-reactive protein in comparison with survivors. 4. C-reactive protein was also reportedly higher [38.9 mg/L (14.3-64.8)] in unstable patients as compared to stable patients [38.9 mg/L (14.3-64.8)], p<0.024.	1.Prospective cohort study/China. 2.Retrospective cohort study/China. 3.Retrospective cohort study/China. 4.Retrospective cohort study/China.
COAGULATION PROFILE	1.Tang N, et al. 2.Zhang L, et al. 3.Zhou F, et al.	1. Non-survivors COVID-19 patients had a higher level of D-dimers, prothrombin time and fibrin degradation products (FDPs) as compared to the survivors. 2. Higher levels of D-dimers were associated with an increased mortality in patients with COVID-19, p<0.001. 3. Non survivors COVID-19 patients had higher level of D-dimers [5·2μg/mL(1·5–21·1)] as compared to survivors [0·6μg/mL (0·3–1·0)], (p<0.0001).	1.Retrospective cohort study/China 2.Retrospective cohort study/China. 3.Retrospective cohort study/China.
CEREBROVASCULAR COMORBIDITIES	1.Mao L, et al. 2.Tralhao A, et al. 3.Avula A, et al. 4.Lodigiani C, et al. 5.Yang X, et al. 6.Madjid M, et al. 7.Aggarwal G, et al.	1. Patients with more severe infection had neurologic manifestations, including acute cerebrovascular diseases (5.7%). 2. The incidence of acute stroke was 0.71% of pooled inpatients of COVID-19. 3. Four COVID-19 patients with acute stroke as a presenting symptom. 4. 2.5% of patients admitted to the hospital with laboratory-proven COVID-19 were diagnosed with ischemic stroke. 5. Comorbidities among the 2 groups of survivors vs non-survivors were 40% (20% vs53%), with cerebrovascular disease (0% vs 22%). 6. Cerebrovascular disease was associated with mortality of COVID-19 patients. 7. 2.5-fold increase in odds of severe COVID-19 illness with a history of cerebrovascular disease.	1.Retrospective cohort study/China. 2.Meta-analysis. 3.Case series/China. 4.Retrospective cohort study/Italy. 5.Retrospective, observational study/China. 6.Review article. 7.Meta-analysis.
HYPERTENSION	1.Guan et al. 2.Guan WJ et al. 3.Hu Y, et al. 4.Liu Z, et. al. 5.Zhou F, et al. 6.Li J, et. al. 7.Lippi G, et. al. 8.Ruan Q, et al. 9.Zheng Z, et. al.	1. Hypertension is prevalent in 23.7% of the patients with severe disease as compared to only13.4% of the patient with non-severe disease 2. Hypertension was found to be more common in patients with severe disease (32.7% vs 12.16%) 3.15.6% COVID-19 patients had hypertension. 4. Crude mortality was found to be much more common in patients with hypertension (6.0%) than in patients without underlying co-morbidities (0.9%) 5. Odds of death in COVID-19 patients having hypertension have been reported to be increased by 3.05 (95% CI 1.57-5.92), p=0.001 6. Severe COVID-19 manifestations, including acute respiratory distress syndrome (ARDS) and more in-hospital deaths, are associated with hypertensive patients 21.3% when compared to normotensive 6.5% (p<0.001) 7. Risk of hypertensive patient dying of COVID-19 increases by a factor of 2.42 (OR=2.42, 95% CI = 1.51-3.90). 8. Hypertension results in an increased mortality in COVID-19 patients (43% died vs 28% survived with p-value=0.007) 9. Hypertension is more common in the group with fatal mortality with OR=2.72, 95% CI (1.60, 4.640) and p-value=0.0002.	1.Retrospective cross-sectional study/China. 2.Retrospective cross-sectional study/China. 3.Meta-analysis. 4.Retrospective cross-sectional study/China. 5.Retrospective cohort study/China. 6.Retrospective cohort study/China. 7.Meta-analysis. 8.Retrospective study/China. 9.Meta-analysis.
DIABETES MELLITUS	1.Roncon L, et al. 2.Huang I, et al. 3.Zhou F, et al. 4.Yan Y, et al. 5.Zhu L, et al.	1. COVID patients who had underlying diabetes had a higher odds of death (OR 3.21, 95% CI 1.82–5.64, p<0.0001, I^2^=16%). 2. DM is associated with composite poor outcome (RR 2.38 [1.88, 3.03], p<0.001) with increased mortality (RR 2.12 [1.44, 3.11], p<0.001) 3. Higher mortality (31% versus 14%, p=0.0051) was seen in diabetics with COVID-19 as compared to non-diabetics 4. Survival among diabetics with COVID-19 was poorer when compared to non-diabetics with a hazard ratio (HR) of 1.53 (95% CI 1.02–2.30; p=0.041). 5. All-cause mortality was 7.8% in diabetic patients with COVID-19 versus 2.7% in non-diabetics [HR 1.49 (95% CI, 1.13–1.96; p=0.005)].	1.Meta-analysis. 2.Meta-analysis. 3.Retrospective cohort study/China. 4.Retrospective, observational study/China. 5.Retrospective cohort study/China.

Age

It is well documented that advanced age is a predictor of mortality in patients with COVID-19, especially in those over 65 years of age. Yang et al. [3] reported that non-survivors were older than survivors (mean age=64·6 vs. 51·9, respectively) and were more likely to have chronic medical conditions, compared to survivors. Mehra et al. [4] found that age greater than 65 years was independently associated with an increased risk of in-hospital death (mortality of 10.0%, vs. 4.9% among those less than equal to 65 years of age; odds ratio [OR], 1.93; 95% confidence interval [CI], 1.60 to 2.41). Recently, the results of Hu et al. [5] indicated that age, in patients over 65 years, was a predictor of unfavorable clinical outcomes, including death (OR=3.546; 95% CI=1.626-7.733, p<0.001). Mortality due to COVID-19 pneumonia was higher in people aged >65 years in a multi-center observational study of 109 decedents with COVID-19 pneumonia carried out by Du et al. [6]. A cohort study by Wang et al. [7] found that the mean age of the non-survivor group was remarkably higher than that of the survivor group. Wang et al. [8], using a multivariate Cox proportional hazards regression model, showed that higher hazard ratios (HRs) for 15-day in-hospital death were associated with age >65 years. Nikpouraghdam et al. [9], in Iran, also demonstrated that for each one-year increase in age, the odds of death increased by 5% (OR=1.05; 95% CI=1.04−1.06). Similarly, Leung et al. [10] found a significant high mortality ratio in COVID-19 patients aged ≥80 years. A quantitative evidence synthesis of clinical and laboratory factors in 852 patients, from Martins-Filho et al. [11], showed an increased risk of in-hospital death in older patients. 

Hematological biomarkers

Ruan et al. [12] found that there were significant differences in total white blood cell (WBC) counts, absolute values of lymphocytes, and platelets between the non-survivor and survivor groups, from a retrospective multicenter study in Wuhan, China. In comparison to non-survivors, there was significant higher lymphocyte count in survivors. In addition, Zhou et al. [13] found that the lymphocyte count was lowest on day seven after onset of illness and improved during hospitalization. Severe lymphopenia was also observed in non-survivors. The most influential marker of risk, according to Wang et al. [7], was severe lymphopenia (<0.5 × 109/L), which was associated with higher mortality (HR=4.410) compared to patients with a lymphocyte count >0.5 × 109/L. Recently, a retrospective review of 323 hospitalized patients with COVID-19 in Wuhan, conducted by Hu et al. [5], indicated that leukocytosis (>10 x109/L, p<0.001) and neutrophilia (>75 × 109/L, p<0.001) predicted unfavorable clinical outcomes. Similarly, in comparison to the survivor group, the non-survivor group was found to have high higher total WBC and neutrophil counts and lower lymphocyte counts as reported by Wang, et al. [8]. Liuet et al. [14] reported that there was an 8% higher risk of in-hospital mortality in patients with increased neutrophil-to-lymphocyte ratio (NLR), and patients in the highest NLR had a 15.04-fold higher risk of death. A meta-analysis from Henry et al. [15] also concluded that compared to survivors, non-survivors had significantly increased WBC counts and decreased lymphocyte and platelet counts. Quantitative evidence of clinical and laboratory factors in 852 patients, from Martins-Filho et al. [11], also showed that survivors had lower levels of white blood cells and neutrophils. Therefore, increased total WBC counts, as well as increased NLR, decreased lymphocytes, and decreased platelet counts are independent risk factors of in-hospital mortality in COVID-19 patients.

Biochemical markers

Ruan et al. [12] reported that total serum bilirubin, blood urea nitrogen (BUN), serum creatinine (Cr), myoglobin, and cardiac troponin were higher in non-survivors than survivors. In comparison to non-survivors, survivors were found to have low levels of cardiac troponin I, Cr, alanine aminotransferase (ALT), and lactate dehydrogenase (LDH) as reported by Zhou et al. [13]. Serum albumin was lower in non-survivors than in survivors. High-sensitivity cardiac troponin I (>0.04 pg/mL, p=0.02) was reported to predict unfavorable clinical outcomes. Wang et al. [8] also demonstrated that Cr, BUN, ALT, aspartate aminotransferase (AST), LDH, and blood ammonia were significantly higher, whereas GFR and serum albumin were significantly lower in the non-survivor group. A meta-analysis by Henry et al. [15] also found that non-survivors had significantly elevated levels of cardiac troponin, liver enzymes (ALT and AST), and renal biomarkers (BUN, Cr) at presentation. Martins-Filho et al. [11] also found that non-survivor patients had increased levels of Cr, creatine kinase, high-sensitivity cardiac troponin I, LDH, and decreased levels of albumin. A meta-analysis of 2389 patients from Santoso et al. [16] demonstrated that cardiac injury was associated with higher mortality, which was defined as high-sensitivity cardiac troponin I>99thpercentile.

Inflammatory biomarkers

Inflammation is the body’s innate immune response to various kinds of injury or insult and during this process a number of proteins are released into the bloodstream. Based on current clinical evidence, inflammatory biomarkers such as C-reactive protein (CRP), serum ferritin, procalcitonin, interleukins, and the erythrocyte sedimentation rate (ESR), play key roles as predictors in evaluating severity and mortality in COVID-19 patients [17].

Rong-Hui et al. conducted a prospective study to evaluate the different predictors of mortality in 179 COVID-19 patients and concluded that non-survivors (n=21) had a higher level of C-reactive protein (mean=86.4mg/L-1; range=39.9−105.5) than survivors (n=158) (mean=36.0 mg/L-1; range=19.3−91.0) with p=0.012 [18].

In another study, Wang et al. evaluated the clinical course of 69 patients with COVID-19 and established that peripheral capillary oxygen saturation (SpO2) <90% was associated with a higher rate of erythrocyte sedimentation and CRP in comparison with patients with SpO2 ≥90% (p<0.001) [19].

In a retrospective study, Fei Zhou et al. concluded that non-survivors had a higher level of interleukin 6 (IL-6) (mean=11·0 pg/mL; range=7·5-14·4) compared to survivors (mean=6·3 pg/mL; range=5·0-7·9; p<0.0001). They also found a higher level of serum ferritin (mean=1435.3 µg/L; range=728.9−2000.0) in non-survivors compared with survivors (mean=503.2 µg/L; range=264.0−921.5; p<0.0001) [13]. CRP was also reported to be higher (mean=38.9 mg/L; range=14.3−64.8) in unstable patients, compared to stable patients (mean: 10.6 mg/L; 1.9−33.1; p=0.024), in a study conducted by Lui et al. [20].

In light of the above-mentioned studies, it is evident that high levels of CRP, ESR, IL-6, procalcitonin, and serum ferritin are associated with worse outcomes and increased mortality of patients with COVID-19.

Coagulation profile

Coagulation abnormalities, ranging from a small decrease in platelet count to disseminated intravascular coagulation, are associated with septicemia, as well as multisystem organ dysfunction syndrome (MODS) [21]. Many coagulative abnormalities have also been observed with SARS-CoV-2 infection, and have been proposed to be associated with increased mortality in COVID-19 patients.

Tang et al. concluded, from a retrospective study involving patients with COVID-19, that non-survivors had higher levels of D-dimers and fibrin degradation products (FDPs), and a raised prothrombin time, compared to survivors (p<0.001) [22]. In another retrospective study, Zhang et al. established that high levels of D-dimers are associated with increased mortality in patients with COVID-19 (p<0.001) [23].

Similarly, Zhouet et al. reported that non-survivors had higher levels of D-dimers (mean=5·2μg/mL; range=1·5-21·1) than survivors (mean=0·6 μg/mL; range=0·3-1·0; p<0.0001) [13].

Cerebrovascular co-morbidities

It has been reported that pre-existing cerebrovascular disease, and stroke after diagnosis of COVID-19, are both predictors of mortality, which might be related to hypercoagulable state from clotting variables and platelet count [24,25,26]. Neurological manifestations including acute cerebrovascular diseases (5.7%), were found more in patients with severe condition with COVID-19 as reported Mao et al. [27]. A meta-analysis of observational studies from Tralhao et al. [28] reported that the incidence of acute stroke was 0.71% in pooled inpatients. Avula et al. [29] reported a series of four COVID-19 patients with acute stroke as a presenting symptom. Lodigiani et al. [30] found that 2.5% of patients admitted to the hospital with laboratory-proven COVID-19 were diagnosed with ischemic stroke. In a single-centered, retrospective, study conducted by Yang et al. [3], the mortality rate was 61.5% by four weeks in 52 critically ill patients with COVID-19. From the review of Madjid et al. [31], cerebrovascular diseases were found to be one of the most important factors associated with mortality in patients with COVID-19. A pooled analysis of published literature from Aggarwal et al. [32], with a sample of 1829 confirmed COVID-19 patients, showed a ∼2.5-fold increase in odds of severe COVID-19 illness with a history of cerebrovascular disease. While a trend was observed, there was no statistically significant association between stroke and mortality in patients with COVID-19 infection.

Hypertension

Hypertension is the single most prevalent comorbid condition in COVID-19 patients, as shown in a study from China where 15% of the patients had hypertension [33]. Similarly, a nationwide survey, a meta-analysis, and a cross-sectional study from China showed that 16.9% [34], 15.6% [35], and 12.6% [36] of the COVID-19 patients had hypertension. However, two other studies showed that 30% [13] and 30.7% [37] had hypertension. These results are not surprising, as this proportion is quite similar to the general incidence of hypertension observed in the general Chinese population, which is 23.2% [38].

Many studies have shown that hypertension is a risk factor associated with increased ICU admission and mortality in COVID-19 patients. Hypertension was found in higher proportion in patients with severe COVID-19 (23.7%) in comparison to non-severe (13.4%) as showed by Guan et al. [33]. Hypertension was also associated with poor composite outcomes included in this study (35.8% vs. 13.7%). Similarly, in another study, hypertension was more common in patients with severe disease (32.7% vs.12.6%) and hypertensive patients were found to have severe outcomes (invasive ventilations or death) in comparison to non-hypertensive (19.7% vs. 5.9%) [34]. Crude mortality was much more common in patients with hypertension (6.0%) than in patients without underlying comorbidities (0.9%) [36]. More severe COVID-19 manifestations, including acute respiratory distress syndrome (ARDS) and in-hospital death, are associated with hypertensive patients (21.3%) than with normotensive patients (6.5%, p<0.001) [37]. A pooled analysis of 13 studies and 2893 patients concluded that the risk of hypertensive patients having severe COVID-19 was 2.5 times higher (OR=2.49; 95% CI=1.98−3.12). Similarly, the risk of hypertensive patients dying of COVID-19 also increases 2.42-fold (OR=2.42; 95% CI=1.51−3.90) [39]. In an article stating that hypertension resulted in increased mortality in COVID-19 patients, 43% died and 28% survived (p=0.07) [12]. A meta-analysis showed that hypertension is more common in the critical/mortality group (OR=2.72; 95% CI=1.60−4.640; p=0.0002) [40]. Odds of death in COVID-19 patients have been reported to be increased 3.05-fold by having hypertension (95% CI=1.57−5.92; p=0.001) [13].

Diabetes mellitus

Diabetes mellitus, characterized by hyperglycemia and insulin resistance, is a chronic inflammatory condition that leads to increased end products of glycosylation, inflammatory cytokine production, and oxidative stress, resulting in tissue damage and increased risk of infections, and leading to morbidity and mortality [41,42,43]. People with diabetes are prone to respiratory tract infections such as influenza and pneumonia [44], and possibly COVID-19. Chronic inflammation, immune response impairment, increased coagulation activity, and (possibly) direct pancreatic damage caused by SARS-CoV-2 are potential underlying mechanisms [45].

In a study conducted on 1382 COVID-19 patients, diabetic patients were found to have increased risk of ICU admission (OR=2.79; 95% CI=1.85-4.22; p<0.0001; I2=46%) and also had a higher probability of death (OR=3.21; 95% CI=1.82-5.64; p< 0.0001; I2=16%) [46].In a meta-analysis of 6452 patients from 30 different studies, Huang et al. concluded that diabetic COVID-19 patients were associated with increased mortality (relative risk [RR]=2.12 [1.44, 3.11]; p<0.001), acute respiratory distress syndrome (RR=4.64 [1.86, 11.58]; p=0.001), and disease progression (RR=3.31 [1.08, 10.14]; p=0.04) [47]. Similarly, a significantly higher mortality (31% versus 14%; p=0.0051) was seen in patients with diabetes with COVID-19 than in those without diabetes in a retrospective cohort study conducted in Wuhan [13].

In a retrospective, observational study, Yan et al. established that survival among those with DM was poorer, with COVID-19, compared to a non-DM group, with a hazard ratio (HR) of 1.53 (95% CI=1.02-2.30; p=0.041), after adjustment for age, sex, hypertension, cardiovascular disease, and cerebrovascular diseases [48]. Moreover, Zhu et al., in a retrospective study, found that all-cause mortality was 7.8% in DM patients with COVID-19 versus 2.7% in non-DM patients (HR=1.49; 95% CI=1.13-1.96; p=0.005). Additionally, all-cause mortality was lower (1.1%) in patients with tight glycemic control than in those with poorly controlled blood glucose levels (11.0%) [49].

In conclusion, the existing literature is clearly showing increased mortality in diabetic patients compared to non-diabetic, especially in patients of advanced age. There is a need to develop novel ways to deliver healthcare to patients with DM using tele-health and for remote patient monitoring for tight glycemic control, without exposing them to unnecessary hazards.

## Conclusions

From the aforementioned literature as summarized in Table 2, we can conclude that the most prevalent risk factor is hypertension followed by diabetes. In terms of mortality, age greater than 65 was the most significant. Amongst non-survivors coagulation profile including d-dimers, prothrombin time, and inflammatory markers like ESR, CRP, and serum ferritin levels were much deranged. Patients with diabetes mellitus, hypertension, cerebrovascular accidents, and age greater than 65 must be in particular managed at home in order to decrease unnecessary exposure at the hospital. From the above data we recommend that the patients with COVID-19 should initially be stratified and segregated on the basis of the above risk factors. Much emphasis and consideration in relation to risk factors must be deliberated by health care providers so as to prevent severe outcomes and mitigate appropriate treatment modalities.
